# Aberrant Methylation-Mediated Suppression of APAF1 in Myelodysplastic Syndrome

**Published:** 2017-04-01

**Authors:** Farhad Zaker, Nahid Nasiri, Naser Amirizadeh, Seyed Mohsen Razavi, Marjan Yaghmaie, Ladan Teimoori-Toolabi, Ali Maleki, Masoumeh Bakhshayesh

**Affiliations:** 1 Cellular and Molecular Research Center, Iran University of Medical Sciences, Tehran, Iran; 2 Dept. of Hematology, School of Allied Medicine, Iran University of Medical Sciences, Tehran, Iran; 3 Blood Transfusion Research Center, High Institute for Education and Research in Transfusion Medicine, Tehran, Iran; 4 Hematology and Oncology Department, Firoozgar Hospital, Iran University of Medical Sciences, Tehran, Iran; 5 Hematology, Oncology and Stem Cell Transplantation Research Center, Tehran University of Medical Science, Tehran, Iran; 6 Molecular Medicine Department, Biotechnology Research Center, Pasteur Institute of Iran, Tehran, Iran; 7 Dept of Hematology, School of Allied Medicine, Tehran University of Medical Sciences, Tehran, Iran

**Keywords:** HRM, Methylation, Myelodysplastic syndrome, APAF1

## Abstract

**Background: **Myelodysplastic syndromes (MDSs) include a diverse group of clonal bone marrow disorders characterized by ineffective hematopoiesis and pancytopenia. It was found that down regulation of APAF1, a putative tumor suppressor gene (TSG), leads to resistance to chemotherapy and disease development in some cancers. In this study, we investigated the relation of APAF1 methylation status with its expression and clinicopathological factors in myelodysplastic syndrome (MDS) patients.

**Materials and** **Methods: **Methylation Sensitive-High Resolution Melting Curve Analysis (MS-HRM) was employed in studying the methylation of CpG islands in the APAF1promoter region in MDS. Gene expression was analyzed by using real time RT-PCR.

**Results:** 42.6% of patient samples were methylated in promoter region of APAF1analyzed, while methylation of the gene was not seen in controls (P<0.05). Methylation of APAF1was significantly associated with the suppression of its mRNA expression (P=0.00). The methylation status of APAF1in advanced-stage MDS patients (80%) was significantly higher than that of the early-stage MDS patients (28.2%) (P=0.001). The difference in frequency of hypermethylatedAPAF1 gene was significant between good (37.5%) and poor (85.71%) cytogenetic risk groups (P=0.043). In addition, a higher frequency of APAF1hypermethylation was observed in higher-risk MDS group (69.2%) compared to lower-risk MDS group (34.14%) (P=0.026).

**Conclusion:** Our study indicated that APAF1hypermethylation in MDS was associated to high-risk disease classified according to the IPSS, WHO and cytogenetic risk.

## Introduction

 MDSs, a heterogeneous group of clonal stem cell disorders, are characterized most commonly by a hypercellular bone marrow, cytopenias and progression to acute myeloid leukemia (AML)^1^^,^^2^.Hypermethylation of CpG islands located in the promoter regions is known to be a frequent and early event in carcinogenesis that regulates gene expression, including expression of tumor suppressor genes (TSGs) and apoptotic genes.^3^^,^^4^There is increasing evidence that aberrant methylation of DNA is one of the underlying mechanisms in pathogenesis of MDS and leukemic transformation.^5^It has been shown that deregulation of apoptosis results in irregular cell survival and has been implicated in the development of cancer. Apoptotic protease activating factor1 (APAF1),a putative TSG, encodes one of the important cytoplasmic proteins in DNA damage – induced apoptosis and is therefore essential for tumor suppression.^6^^,^^7^ In apoptotic cascade, cytochrome C released from the mitochondria in response to cell death stimulus is bound to APAF1in the cytosol. APAF1 in association with ATP is released from auto-inhibited state and would be bound to procaspase.^9^Thus, ATP binding allows oligomerization of APAF1, which is necessary for auto-activation of caspase-9, successive activation of downstream procaspases and eventual programmed cell death (apoptosis).^8^^–^^10^ In addition, APAF1 is recognized as an essential downstream effector of p53-mediatedapoptosis. ^11^^,^^12^  Given the role of *APAF1* in apoptosis, it seems that its inactivation plays a role in oncogenic transformation and drug resistance. ^13^^–^^15^ 

In this study, we aimed to elucidate methylation status of CpG islands in the *APAF1 promoter *region in MDS patients. In addition, the association between the methylation status of APAF1 and its expression with clinicopathological variables of patients was also evaluated. To conclude then, in this study we have demonstrated elevation in hypermethylationofAPAF1 and subsequently suppression of its expression in advanced-stage MDS, indicating that APAF1 might be involved in disease progression.

**Table 1:** Correlation betweenAPAF1 methylation and clinical features in MDS patients

**Characteristics**	***APAF1*** ** Methylated (23)**		***APAF1*** **Unmethylated (31)**		**P-value**
** Median (range)**		n (%)	Median( range)	n (%)	
**Age (Years)**	63.09(23-90 )		60.42(38-87)		0.501
**Sex ** ** Male** ** Female **		8(27.56%) 15(60%)		21(72.44%) 10(40%)	0.016
**WBC (×109/L)**	4.5(1.3-10)		5.2(1.2-13)		0.321
**ANC (×109/L)**	2.2(0.29-4.4)		3(0.59-8.4)		0.057
**Hb(g/dL)**	9.32(5.6-14.8)		9.86(5.8-14.3)		0.33
**Platelets (×109/L)**	113.8(7-752)		141.9(9-383)		0.34
**SF ng/ml**	357(55-846)		408(2.9-1600)		0.564
**LDH U/L**	522(120-1886)		326(99-732)		0.02
**LDH>400U/L** **LDH<400U/L**		14(63.63%) 9(28.1%)		8(36.36%) 23(71.9%)	0.01
**BM Blast (%)** **<5%** **>5%**		11(28.2%) 12(80%)		28(71.8%) 3(20%)	0.001
**WHO** ** Early stage** **( RA, RT, RCMD, 5q-)** ** Advanced stage** **(RAEB-1, RAEB-2)**		11(28.2%) 12(80%)		28(71.78%) 3(20%)	0.001
**WHO** ** RA (n=20, 37%)** ** RT (n=6, 11.1%)** ** RCMD (n=10, 18.5%)** **RAEB-1 (n=4, 7.4%)** ** RAEB-2 (n=11, 20.4%)** ** -5q (n=3, 5.6%)**		7(35%) 1(16.67%) 2(20%) 4(100%) 8(72.72%) 1(33.33%)		13(65%) 5(83.33%) 8(80%) 0(0%) 3(27.27%) 2(66.66%)	0.017
**IPSS** ** Low risk (Low/Int-1)** ** High risk (Int-2/High) **		14(34.14%) 9(69.2%)		27(65.86%) 4(30.8%)	0.026
**IPSS-R ** ** Very low** ** Low ** **Int** ** High** ** Very high**		6(30%) 2(15.38%) 5(62.5%) 3(75%) 7(77.77%)		14(70%) 11(84.61%) 3(37.55%) 1(25%) 2(22.22%)	0.009
**Cytogenetic** ** Normal karyotype** ** Abnormal karyotype**		10(37%) 13(48.14%)		17(63%) 14(51.85%)	0.409
**Karyotype(IPSS) ** ** Good** ** Intermediate ** ** Poor **		15(37.5%) 2(28.6%) 6(85.7%)		25(62.5%) 5(71.4%) 1(14.3%)	0.043
**Karyotype(IPSS-R) ** ** Very Good** ** Good ** ** Intermediate** ** Poor** ** Very poor **		0(0%) 15(41.66%) 2(25%) 3(100%) 3(100%)		4(100%) 21(58.33%) 6(75%) 0(0%) 0(0%)	0.017

WBC indicates white blood cell count; ANC, absolute neutrophil count; Hb, hemoglobin; Plt, platelet; SF, serum ferritin; LDH, lactate dehydrogenase; IPSS-R, international prognostic scoring system (revised); WHO, world health organization; RA, refractory anemia; RT, refractory thrombocytopenia; RCMD, refractory anemia with multilineage dysplasia ; RAEB-1, refractory anemia with excess blasts-1; REAB-2,refractory anemia with excess blasts-2; -5q, MDS associated with isolated del (5q-).

## Materials and Methods

**Patients and Sa** **mples**

The research was approved by the Ethics Committee of Iran University of Medical Sciences. Sixty patients diagnosed with MDS in Shariati Hospital and Firouzgar Hospital (Tehran, Iran) were enrolled in this study between 2012 and 2014. Informed consent was obtained from all donors. Because of insufficient data, six patients were excluded from this study. To assess the clinical impact of APAF1 methylation, we have analyzed the variables listed in Table 1. Mononuclear cells were isolated from bone marrow aspirates or peripheral blood of patients and healthy donors by sedimentation on Ficoll-Paque PLUS** (GE Healthcare Life Sciences).** Controls used in experiments were DNA from 20 healthy donors (median age of 62.45 years) and EpiTect®PCR Control DNA (Qiagen, Hilden, Germany).

WBC indicates white blood cell count, ANC, absolute neutrophil count, Hb, hemoglobin, Plt, platelet, SF, serum ferritin, LDH, lactate dehydrogenase, IPSS-R, international prognostic scoring system (revised), WHO, world health organization, RA, refractory anemia, RT, refractory thrombocytopenia, RCMD, refractory anemia with multilineage dysplasia , RAEB-1, refractory anemia with excess blasts-1, REAB-2,refractory anemia with excess blasts-2, -5q, MDS associated with isolated del (5q-).

**Conventional** **C** **ytogenetic Analysis**

Conventional cytogenetic analysis was carried out on marrow aspirates. Karyotypes were analyzed on banded metaphases. Chromosome abnormalities were described according to the International System for Human Cytogenetic Nomenclature (ISCN).^16^

**Bisulfite** **C** **onversion and MS-HRM**

Genomic DNA was isolated using QIAamp DNA Blood Mini Kit (Qiagen, Hilden, Germany) according to the manufacturer’s guidelines. Genomic DNA (1000 ng) was bisulfate modified, using the EpiTect Bisulfite Kit (Qiagen, Hilden, Germany) according to the manufacturer’s protocols. 

MS-HRM was run in a volume of 10 μl, containing 20 ng of converted DNA, 5 μl of EpiTect HRM-PCR Kit (Qiagen, Hilden, Germany) and 2.5 pmol of each primer. Primers used were as follows: for *APAF1*, F: 5′- ATTYGAGGAGGAGGGGTAGGA-3′and R: 5′- GCT TAT ACA AAT AAA CAC ACC CCA AAC -3′ (228 bp). PCR conditions were carried out in a rotor gene TM 6000 device (Corbett Research, Mortlake, Australia) as follows: a 95°C hot start for 5 minutes, 95°C for 10 seconds, 62°C for 30 seconds and 20 seconds at 72°C for 40 cycles. Following gene amplification, the HRM was performed from 56°C to 99°Cand the temperature was increased by 0.1°C / 2 s. **RNA Extraction, cDNA Synthesis and Fluorescence Quantitation RT-PCR **

RNA was extracted from mononuclear cells (MNCs) by TriPure Isolation Reagent (Roche Applied Science, Penzberg, Germany) according to themanufacturer'sguidelines.ThecDNAwassynthesizedusingQuantiTect Reverse Transcription Kit (Qiagen, Hilden, Germany) according to the manufacturer’s instructions. qPCR was performed with QuantiFast SYBR Green RT-PCR Kit (Qiagen, Hilden, Germany) on rotor gene 6000 device. 

Each reaction was carried out in a final volume of 10 μl as follows: 5 μl of 2 × SYBR Green master mix, 2 μl of cDNA and 2.5 pmol of each primer. Glyceraldehyde-3-phosphate dehydrogenase (GAPDH) was used to normalize RNA levels. Primers used were as follows: for *APAF1*, F: 5′-GGCTGTGGGAAGTCTGTATTAGC-3′ and R: 5′-ACTCTCATCCTGATCCAACCG-3′ (165 bp);for GAPDH, F: 5′-CACCAGGGCTGCTTTTAACTCTGGA-3′ and R: 5′-CCTTGACGGTGCCATGGAATTTGC-3′ (130 bp).The amplification conditions were as follows: 5 minutes at 95 °C, 40 cycles for 10 seconds at 95 °C and 30 seconds at 60 °C (combined annealing/extension step). 

**Statistical Analysis**

We applied Mann–Whitney’s U-test, Kruskal-Wallis test, ANOVA, Student’s t-test and Chi- square test. All statistical analyses were performed using the SPSS 16.0 software package (SPSS, Chicago, IL). P values ≤0.05 were considered significant.

## Results

**Patient’s** **Ch** **aracteristics**

29 males and 25 females with a median age of 61 years (range, 23–90 years) were enrolled in this study. MDS diagnosis was made based on WHO criteria. Clinical and demographic data were summarized in Table 1.

**Correlation between** **Gene Methylation and Expression in MDS Patients**

Quantitative *Real-time RT-PCR *was done to determine whether the promoter methylation was related tomRNAdownregulation.APAF1 promoter methylation was shown to be inversely correlated with the mRNA expression in patients (*P*=0.00).There was a significant difference in the delta Ct of the *APAF1* mRNA between patients and normal subjects/controls using the two-tailed *t*-test (P= 0.007). Delta Ct average was significantly greater in patients with advanced-stage MDS (average delta Ct 3.6) compared with early-stage MDS (average delta Ct 2.6) or controls (average delta Ct 1.6). The difference in the delta Ct between advanced-stage MDS and controls was statistically significant (*P*=0.001).Gene expression average in early-stage MDS was higher than that in advanced-stage MDS and this difference was statistically significant (2.62 vs. 0.46, P=0.03). The highest and the least average in APAF1 fold change was observed in RCMD and RAEB-1, respectively (4.14 vs. 0.11, respectively). We discovered that APAF1expressionfoldchangewashigherinlow-risk MDS than that in high-risk MDS (2.23 vs. 1.69, respectively), but it was not statistically significant (*P*=0.535). In further analysis, the correlation between gene expression and IPSS score was statistically significant (P=0.02). No significant differences were observed between *APAF1* fold change and WBC, ANC, platelets, Hb, age, SF and LDH levels, cytogenetic risk groups and IPSS-R in MDS patients (P>0.05). 

**APAF1Promoter Was Hypermethylated in Patients with MDS**

**Fig 1:**
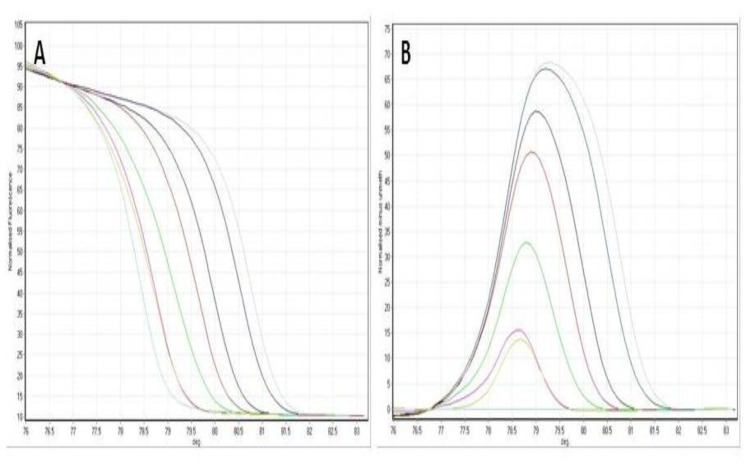
APAF1 HRM curves for methylation standards containing varying amounts of methylated DNA. (A)Normalized graph for APAF1. (B) Difference plot for the data represented in (A).Standards 100% gray line, 90% dark blue line, 75% black line, 50% red line, 25% green line, 10% purple lines, 0% blue line, yellow line for patient sample.

DNA methylation was measured with the MS-HRM. Standard curves with commercial controls were drawn for validation of HRM (Figure 1).

**Fig 2:**
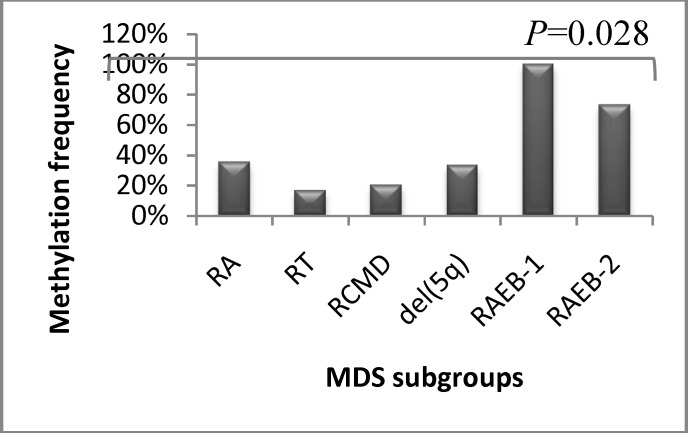
Methylation frequency in MDS subgroups. The number of patients with hypermethylated APAF1 increased from early-stage MDS (RA, RT, RCMD, del [5q] to advanced-stage MDS (RAEB-1, RAEB-2) (P=0.028).

Aberrant DNA methylation of APAF1 gene (range, 1% - 18%) in MDS and control groups was 42.6% (n= 23/54) and 0% (n=0/20), respectively, showing the difference was significant (P<0.05). MeanAPAF1 methylation was higher in advanced-stage MDS (RAEB-1/RAEB-2) group of patients (12 out of 15 cases(80%), x^2^=2.93) compared with early-stage MDS (RA/RCMD/5q- syndrome) group (11 out of 39 cases(28.2%), x^2^=0.97) (*P<*0.01).Methylation frequency of APAF1 gene in MDS subgroups is shown in Figure 2. 

Frequency of hypermethylated*APAF1* gene was statistically different among IPSS-R prognostic risk categories (P=0.009). Average methylation level of APAF1gene in IPSS low/Int-1 risk group was significantly lower than that in the IPSS Int-2/high risk group (1.24 vs. 2.38, respectively).Methylation frequency ofAPAF1 gene was statistically significant between good and poor cytogenetic risk groups (*P*<0.05).No significant differences were observed between the hypermethylated and non-hypermethylated MDS patients in WBC, ANC, platelets, Hb, age, and SF level (P>0.05). Moreover, there was a trend that the higher-risk group had a higher LDH level, age and blast count in comparison with the lower-risk group (P=0.006, P=0.016, P=0.00, respectively). There was no significant difference among other parameters.

## Discussion

 Apoptosis is one of the main pathways often deregulated in MDS.^17^There is increasing evidence that during early course of MDS disease, increased apoptosis is associated with decreased cell survival, while during later phases, the overexpression of pro survival proteins, decreases apoptotic rate of bone marrow cells and promotes the possibility of the evolution of MDS syndromes to AML.^17^^,^^18^ 

The aberrant methylation of genes involved in the apoptotic pathway is poorly defined in MDS. When aberrant methylation occurs in the promoter of genes that suppress tumorigenesis, it can lead to the resistance to apoptosis and progression of cancer^19^.Since the*APAF1*gene, a TSG, is rarely mutated, promoter hyper methylation appears to be the mechanism underlying its inactivation.^20^In this study, we examined expression of APAF1 in MDS and found that APAF1 mRNA level was diminished in 53.7% of MDS cells. Our data indicated a higher level of APAF1 transcripts in low-risk MDS compared to the high-risk disease as categorized based on WHO and IPSS (P<0.05). No difference was observed when the groups were compared according to the cytogenetic risk score (P>0.05). Consistent with other investigations, a decreased expression of APAF1 in high-risk MDS may be indicative of a role for APAF1 in MDS progression or lower levels of apoptosis in high-risk MDS compared to low-risk MDS. It has been shown that increased expression of APAF1in low-risk disease and its positive correlation with the apoptotic rate may be indicative of APAF1rolein the pathophysiology of MDS.^21^

Aberrant DNA methylation has been associated with downregulation of genes.^22^^,^^23^

The relationship between prognosis and DNA methylation has been studied in MDS on a single candidate gene or combinations of multiple genes. For instance, a study of patients with MDS showed that concordant methylation ofmultiple genes predicts poor prognosis and risk of leukemia transformation.^24^Our analysis of*methylation status of APAF1*gene promoter in patients with MDS showed thatAPAF1hypermethylation frequency was 42.6%. In this study,we presented evidence for elevation of APAF1methylationin MDS patients during the progression from low-risk (28.2%) to the high-risk disease (80%). An association of methylation-associated silencing of *APAF1* expression with higher IPSS-R score and advanced-stage MDS was recorded. Our results suggest that *APAF1* may be implicated in the acquisition of a more aggressive phenotype in MDS. Cytogenetic analysis is one of important risk factors for predicting leukemic evolution.^25^ In addition, a recent study provides new information about the role of cytogenetic analysis in diagnosis, prognosis and follow-up of patients with hypocellular primary MDS.^26^ In our analysis, a correlation with chromosomal aberrations was elicited for *APAF1* promoter methylation. These results may indicate that hyper methylation may contribute to the leukemogenesis. LDH is a useful prognostic parameter in several hematological malignancies.^27^^-^^29^We discovered the association betweenAPAF1 hypermethylation and initial LDH level to a statistically significant extent. In further analysis, there was a strong correlation between cytogenetic risk categories, MDS subgroups and IPSS-R with level of LDH activity. High *LDH level* remained a significant adverse prognostic factor for high-risk patients. The results showed that APAF1 promoter hypermethylation was correlated closely with the loss of APAF1 mRNA expression, indicating that function of this gene may recover following demethylation. The present study, along with previous reports^30^, determines APAF1 as another target of methylation silencing. The evidence has been shown that demethylation treatment can restore expression of APAF1 at both mRNA and protein levels and, therefore, activate apoptotic pathway.^31^In another study, Furukawa et al. showed overexpression of Dnmt1 mediatedAPAF1 gene methylation that was reversed by demathylation agents.^20^ In the present study, following statistical analysis, the expression and hypermethylation of APAF1 was not significantly correlated with the age, gender, hematologic variables and SF of the patients (P>0.05). 

In conclusion, the present study is consistent with some observations in oncogenesis that have identified the loss of APAF1 as a substantial characteristic in development of tumor. With the increased progression of MDS, the expression of APAF1 mRNA tends to decrease. Gene silencing following methylation is an important epigenetic mechanism of gene down- regulation. Furthermore, our results imply that hypermethylation is associated to high-risk disease as classified according to the IPSS, WHO, and cytogenetic risk. Therefore, APAF1 may serve as prognostic indicator in advanced-stage MDS. Our results also confirm baseline LDH as a significant prognostic marker.

## CONCLUSION

 In summary, the present study is consistent with some observations in oncogenesis that have identified the loss of APAF1 as a substantial characteristic in development of tumor. With the increased progression of MDS, the expression of APAF1 mRNA tends to decrease. Gene silencing following methylation is an important epigenetic mechanism of gene down-regulation. Furthermore, our results imply that hypermethylation is associated to high-risk disease as classified according to the IPSS, WHO, and cytogenetic risk. Therefore, APAF1 may serve as prognostic indicator in advanced-stage MDS. Our results also confirm baseline LDH as a significant prognostic marker.
